# Mandibular range of motion in patients with idiopathic peripheral facial palsy

**DOI:** 10.1590/S1808-86942011000200014

**Published:** 2015-10-19

**Authors:** Fernanda Chiarion Sassi, Laura Davison Mangilli, Michele Conceição Poluca, Ricardo Ferreira Bento, Claudia Regina Furquim de Andrade

**Affiliations:** 1 PhD in Sciences - Medical School of the University of São Paulo. Speech and Hearing Therapist, head of the Facial Paralysis Ward - Speech and Hearing Department - University of São Paulo Hospital - FMUSP; 2 PhD student - Rehabilitation Sciences Program - Department of Physical Therapy, Speech and Hearing Therapy and Occupational Therapy-FMUSP. Speech and Hearing Therapist, head of the Orthognathic Surgery Ward - University of São Paulo Hospital - FMUSP; 3 Speech and Hearing Therapist. Specialized in Facial Functions - Permanent Education School - HC-FMUSP; 4 Full Professor - Department of Ophthalmology and Otorhinolaryngology of the Medical School of the University of São Paulo - Director of Otorhinolaryngology - FMUSP; 5 Full Professor - Department of Physical Therapy, Speech and Hearing Therapy and Occupational Therapy - FMUSP. Director of the speech and Hearing Department - Medical School of the University of São Paulo - FMUSP

**Keywords:** facial paralysis, speech, language and hearing sciences, anthropometry

## Abstract

Regarding orofacial motor assessment in facial paralysis, quantitative measurements of the face are being used to establish diagnosis, prognosis and treatment planning.

**Aim:**

To assess the prevalence of changes in mandibular range of motion in individuals with peripheral facial paralysis.

**Materials and Methods:**

Prospective study. We had 56 volunteers, divided in two groups: G1 made up of 28 individuals with idiopathic facial paralysis (6 males and 22 females); 14 with manifestations on the right side of the face and 14 on the left side; time of onset varied between 6-12 months; G2 with 28 healthy individuals paired by age and gender to G1. In order to assess mandibular range of motion, a digital caliper was used. The following measurements were made: 1) middle line; 2) maximum oral opening; 3) lateralization to the right; 4) lateralization to the left; 5) protrusion; 6) horizontal overlap.

**Results:**

Statistically significant differences between the groups were observed for maximum oral opening, lateralization to the left and protrusion. G1 presented smaller measurement values than G2.

**Conclusion:**

Patients with facial paralysis present significant reduction of mandibular range of motion. The results support the suggestion of incorporating functional evaluation of the temporomandibular joint to the existing facial paralysis clinical assessment protocols.

## INTRODUCTION

Facial paralysis is different from most disorders which affect the facial muscles insofar as clinical circumstances are concerned, because the outcome has a variety of symptoms. It may arise from skull base injuries, congenital syndromes, low skull tumors, infectious diseases and others, leading to this unique disability^1^, ^2^, ^3^.

Bell's palsy is a peripheral palsy of the facial nerve, which results from the total or partial reduction in facial muscle mobility. It is traditionally described as idiopathic; nonetheless, a possible etiology could be infection by the type 1 herpes virus. Although Bell's palsy can affect people of any age, its top incidence is on the fifth decade of life. Approximately 70% to 80% of patients recover spontaneously^1^, ^2^, ^4^.

The annual incidence of Bell 's palsy is 15 to 30 for every 100,000 people, and there does not seem to be gender differences. There is no predilection concerning face side^3^. The affected patients develop paralysis on their facial muscles of one and three days of duration, without any other neurological disorder. Usually, symptoms get to a maximum on the first week and then they diminish gradually between three weeks and three months^4^. It happens more frequently in diabetic individuals and in pregnant women. Patients who had one Bell's palsy episode have an 8% risk of recurrence^4^.

Patients with facial palsy usually complain of weakness or complete paralysis of all the muscles on one side of the face. Creases and the nasolabial folds completely disappear and the mouth corner tilts. One common Bell's palsy characteristic is the incomplete closure of the eyelid, resulting in dry eye. The eye irritation frequently results from the lack of lubrication and constant exposure^4^.

Facial paralysis frequently has a significant emotional impact on affected patients^5^. One of the most importantly affected facial movements is the smile, a facial expression which is predominantly used in social communication. The smile is a complex social and emotional expression and failure to smile was a preliminary motivator factor to indicate surgical treatment^6^.

One treatment option is surgical reanimation, which aims at restoring facial symmetry and voluntary control over facial muscles, enabling the patient to express his/ her emotions, besides promoting eye protection and oral continence, thus enhancing quality of life^7^. Although movement can be improved, there is no surgical method which restores spontaneous involuntary movement associated with emotions^5^, ^6^, ^7^, ^8^.

The best known and most used assessment system is the House-Brackmann (HB) score. This scale has 6 grades, which are used to classify the level of facial nerve injury. This score is established by measuring the upper movement of the top of the eyebrow and the lateral movement of the corner of the mouth. It is a scale which establishes the severity of the facial paralysis^9^, ^10^. The speech and hearing therapist's assessment must encompass instruments which enable functional measuring. These measures will enable treatment and help check for treatment efficacy.

The speech and hearing quantitative measures can be obtained through surface electromyography; photogrammetry; digital caliper; cephalometry and, more recently, the facial movement quantification system, in 3-D video^11^, ^12^, ^13^, ^14^, ^15^. The qualitative measures may be obtained through clinical protocols and self-perception questionnaires^16^, ^17^.

In speech and hearing therapy practice concerning facial paralysis patients, it is not a routine to completely assess orofacial functions, because the main complaint is associated with facial movements. Nonetheless, frequent have been the reports of pain in the temporomandibular joint (TMJ) and a reduction in speech articulatory movements. In the bibliography search, we did not find specific studies on the relationship between facial paralysis and TMJ function. The TMJs are important structures of the stomatognathic system, since they enable mandibular movements and functions such as suction, swallowing, chewing and speech^18^.

Literature states that the range of mandibular motion is associated with TMJ integrity and the action of skeletal muscles^19^, ^20^, ^21^. The TMJ needs to support and accommodate occlusal, muscular and neck adaptations. When the demand for functional adaptations exceeds the TMJ functional and structural tolerance, the patient may develop signs and symptoms of temporomandibular disorders (TMD)^22^, ^23^, ^24^, causing changes to mandibular movements and to the stomatognathic functions associated with them^19^. These changes to the mandibular movements often cause complaints of pain, which causes reduction in range of motion, thus affecting speech articulation^21^.

Concerning mouth opening, we notice its reduction in individuals who have TMJ disorders^22^. The risk of otological symptoms is considered high in patients who feel pain upon palpation of their TMJs, masticatory and neck muscles, as well as pain upon mouth opening^22^. Studies report that the main signs/symptoms were: joint noise, muscle pain and TMJ pain; and also, frequently present were: neck pain and teeth pain. Studies found in the literature state that among otological symptoms, ear fullness prevails over ear ache and tinnitus^23^.

Some authors report the association of condyle fossa non-concentric relations to the abnormal TMJ function, as well as others associated with the bilateral symmetry of the condyle and the absence of clinical symptoms in adults. Nonetheless, the role of the condyle position in the TMD etiology is still controversial in the literature^21^. Studies have established the correlation between TMD signs and symptoms and the condyle position in the mandibular fossa.

Sequelae may happen at about four months after facial paralysis ensues, such as contractures and hypertrophy of the facial muscles, in association with synkinesia (independent movements)^25^. Spasms of many areas worsen the condition, and they are mainly located on the eyelids and lip commisure^2^, ^4^. Another possible sequela - TMJ pain - may arise from unilateral chewing and consequent orofacial muscle strength dysbalances^26^.

Our study aimed at assessing the prevalence of changes to the mandibular range of motion (mouth opening; mandible lateralization and protrusion) in patients with idiopathic peripheral facial paralysis.

## MATERIALS AND METHODS

### Participants

The individuals who participated in this study only started the assessment process after the proper ethical procedures. All the data was collected in the ENT Service of the Speech and Hearing Therapy Department of a public hospital of São Paulo.

In this study we had a total of 56 voluntary individuals from both genders, with ages varying between 15 and 61 years. These individuals did not have speech and hearing disorders such as: communications, auditory, neurological and cognitive complaints or deficits, according to medical definitions.

These individuals were divided in two groups: Group 1 (G1), made up of 28 patients with idiopathic facial paralysis (FP), diagnosed in the ENT Ward, six males and 22 females, 14 with right-side facial paralysis and 14 with left-side facial paralysis, with paralysis duration time varying between 6 months and 2 years; Group 2 (G2) was made up of 28 individuals without facial movement disorders, paired in age and gender with those from G1.

G1 inclusion criteria:

a) Individuals with a medical diagnosis of peripheral facial paralysis, without surgical intervention for facial nerve reanimation or reconstruction;
b) Not having a past of facial trauma and/or surgeries on the face or neck;
c) Not having partial or total teeth prosthesis;
d) Having a score between 4 and 11 in the Facial Movement Clinical Assessment Protocol for the Paralyzed Side^27^ (Attachment 1).

G2 inclusion criteria:

a) Not having complaints or medical diagnosis of facial paralysis;
b) Not having a past of facial trauma and/or surgeries on the face or neck;
c) Not using partial or total dental prosthesis;
d) Having a score of 19 or 20 in the Facial Mimicry Clinical Assessment Protocol^27^ (Attachment 1).

### Materials

**Figure 1:**
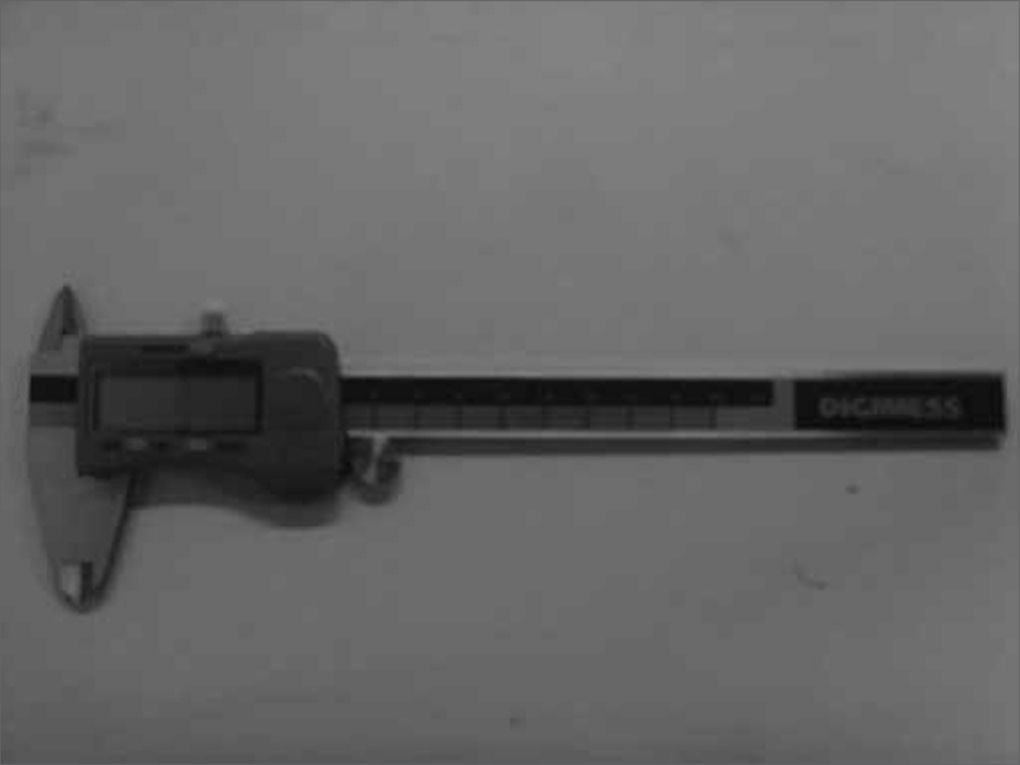
Digimess Pró-Fono Digital Caliper

The present study used the following materials: Sliding-type Pro-Fono Digimess Digital Caliper (Fig. 1), disposable surgical gloves, cotton balls, hydrated ethylic alcohol, Facial Paralysis Clinical Assessment Protocol^27^, Complementary Protocol of mandibular range of motion values.

### Procedure

For the clinical assessment of facial mimicry we used a protocol^27^ which assesses the facial functional/cosmetic symmetry (Attachment 1). The muscle groups from each facial side were analyzed under different voluntary facial expressions, being scored from zero (0) if there were no movements; one (1) for partial or moderate movement, and two (2) for complete or marked movement.

The frontal region was assessed by the movement of eyebrow raising, eyelid move during eye closure, upper lip elevation through the movement of “frowning the nose”, oblique traction of the upper lip required for smiling, horizontal traction of the upper lip by the clinical smile, lip closure by means of lower lip protrusion and depression with the movement for showing the lower teeth.

After this stage, the involuntary emotion-related movements were assessed in each side of the face by observing the participants during blinking, talking and smiling spontaneously, using the same previous scoring criteria, zero (0) when absent, one (1) when reduced and two (2) when normal. Lips and eyelids deformities upon rest, the presence of synkinesia or hypertonia were also scored with negative values, (0) if absent, (-1) if partial or mild deformity and (-2) if total or severe deformity . At the end, the partial sum of the values obtained amounted to the final score, which could range from -6 to 20 points for each evaluated hemiface.

**Figure 2:**
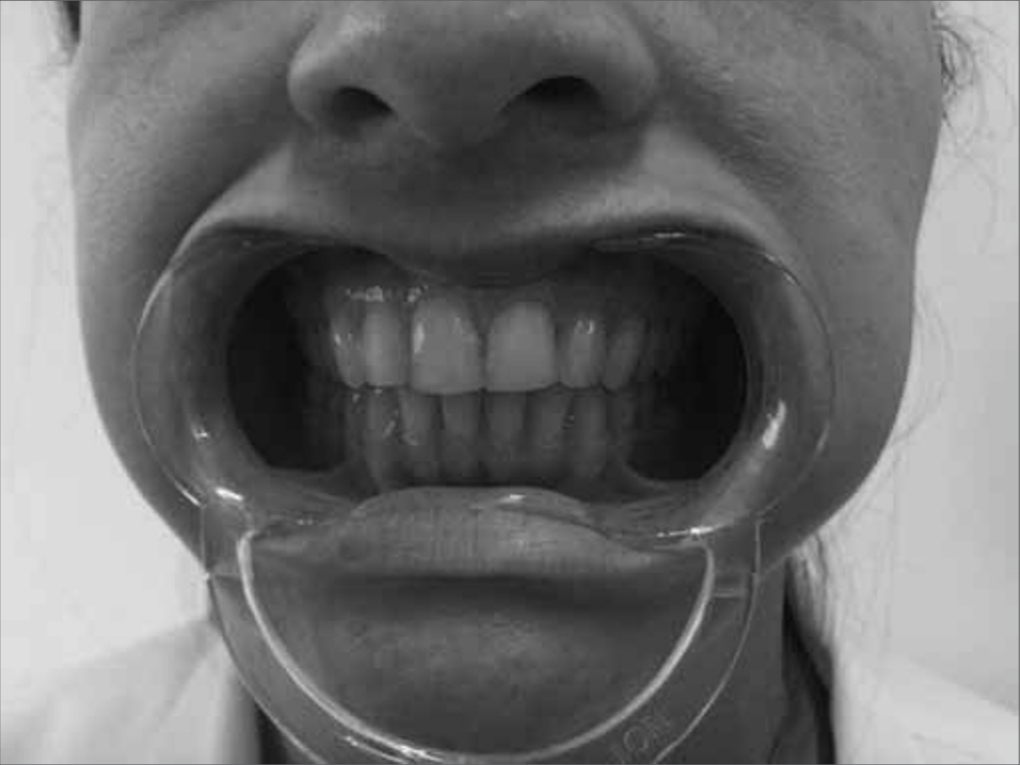
Midline.

**Figure 3:**
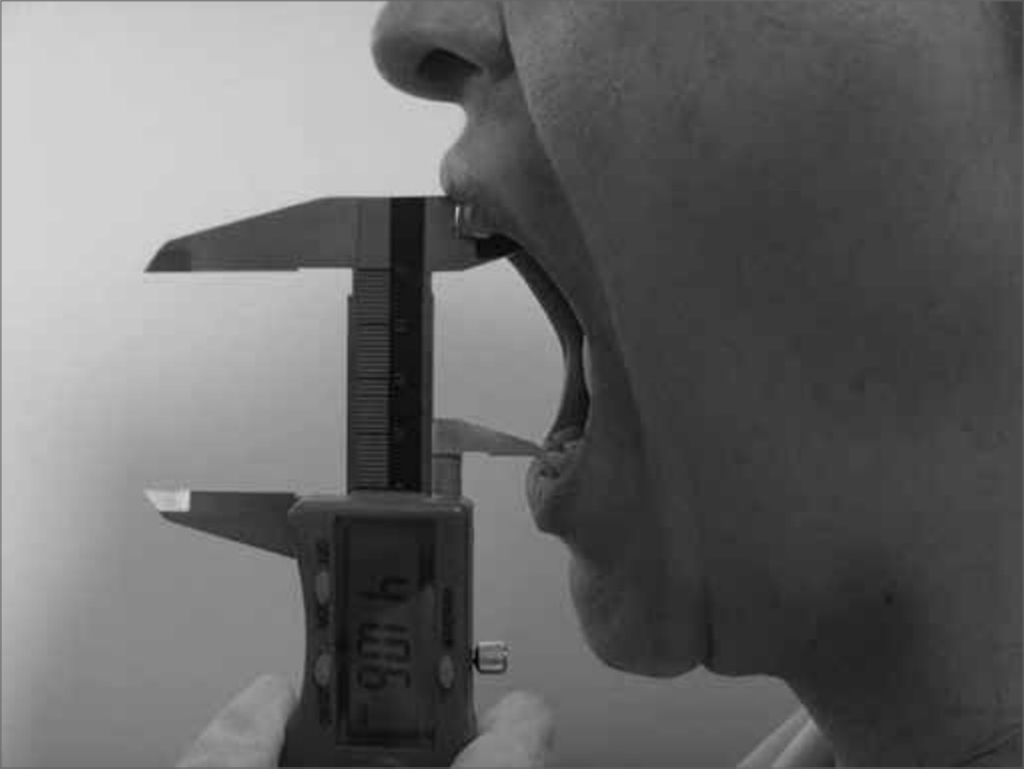
Maximum mouth opening.

**Figure 4:**
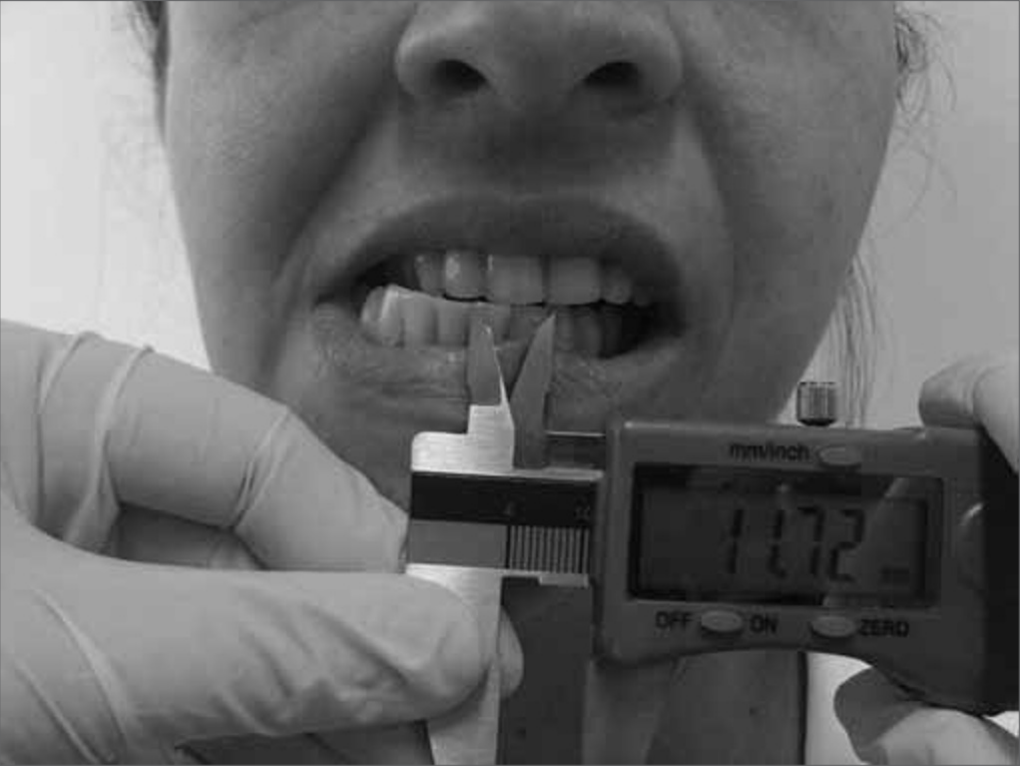
Mandible lateralization to the right.

**Figure 5:**
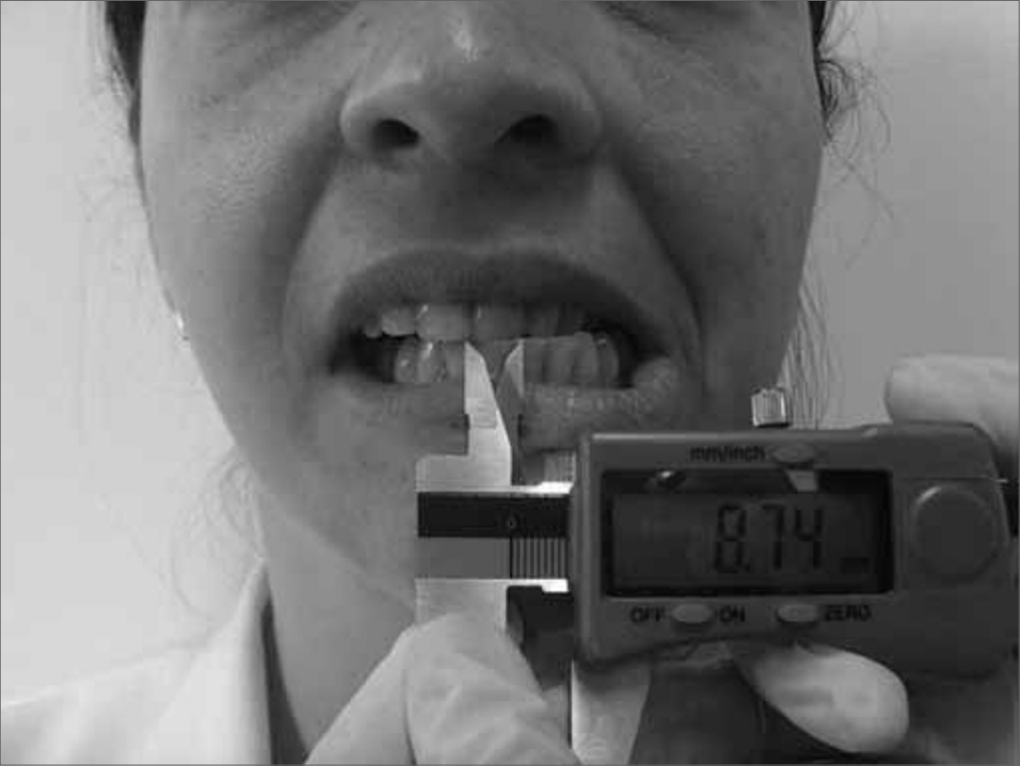
Mandible lateralization to the left.

**Figure 6:**
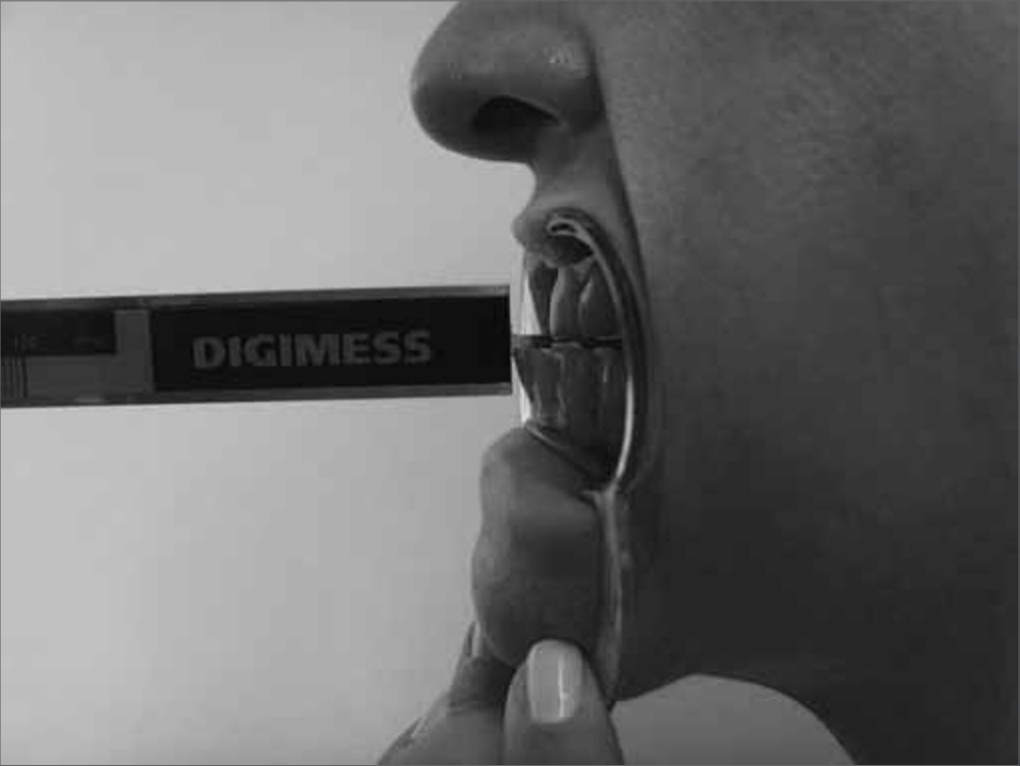
Mandibular protrusion.

The technique used to measure mandible range of motion was based on the methodology proposed by Cattoni^28^ et al. and Ferreira^29^, and Felício & Trawitzki^30^. Using the digital caliper we measured the following mandible movements: 1) mid line - with the teeth in occlusion - we checked whether or not the lines between the central upper and lower incisive teeth matched (Fig. 2). When they did not match, we measured when one line was distant from the other horizontally, that is, a deviation from the mid line; 2) maximum mouth opening - we measured the distance between the incisive faces of the upper and lower teeth and added the vertical trespass value (Fig.3); 3) mandible lateralization to the right - we measured the horizontal distance of the line between the lower central incisive teeth to the line between the upper central incisive teeth after right-side mandible shifting. When there was a midline deviation, we used the pertaining adjustment (Fig. 4); 4) mandible lateralization to the left - the same procedure carried out to measure mandible lateralization to the right was used to obtain the mandible lateralization to the left value (Fig. 5); 5) mandible protrusion - summation of the horizontal trespass value with the maximum horizontal shifting of the mandible (Fig. 6) 6) horizontal trespass - in occlusion - here we measured the distance between the occlusal face of the upper central incisive and the distal face of the lower central incisive. All measures were taken three times and checked by 3 experienced examiners, with an 85% rate of agreement between them.

For each value we did a total of six measurements (three measurements from each speech and hearing therapist). The individuals were positioned in the following way: seating with their feet on the floor, head in the standard position, placed according with the Frankfurt horizontal plane. The examiner was placed in front of the individual in order to take the measure. Should a difference of more than 25% be seen in the results, the value was measured again.

For the statistical analysis of the data we used the ANOVA, confidence interval for the mean and *p*-value tests, with 0.05 significance level (5%).

### RESULTS

Table 1 shows a comparison between G1 and G2 concerning maximum mouth opening.

**Table 1:** G1 and G2 comparison for Maximum Mouth Opening

Opening	G1	G2
Mean	43.25	47.60
Median	43.14	47.04
Standard Deviation	8.62	5.81
VC	20%	12%
Min.	24.75	31.20
Max.	61.55	59.89
N	28	28
CI	3.19	2.15
*p*-value	0.031*	

VC - variation coefficient; Min - minimum value; Max - maximum value; N - number of mean values (participants); CI - confidence interval

Results indicate that the groups are statistically different, G1 had a maximum mouth opening lower than that of G2.

Table 2 shows the group comparison as to right-side mandible shifting. For this first analysis we did not consider the facial paralysis side.

**Table 2:** Comparison between G1 and G2 for the right-side shifting value

R. Shift	G1	G2
Mean	6.37	7.56
Median	6.11	7.96
Standard Deviation	3.02	2.31
VC	47%	31%
Min	1.50	1.42
Max	15.25	11.23
N	27	28
CI	1.14	0.86
*p*-value	0.108	

VC - variation coefficient; Min - minimum value; Max - maximum value; N - number of mean values (participants); CI - confidence interval

We did not find statistical differences for the groups concerning this comparison.

Table 3 shows group comparison as to mandibular shift to the left. For this analysis we also did not consider the facial paralysis side.

**Table 3:** Comparing G1 and G2 concerning the left shifting value.

L. Shift	G1	G2
Mean	6.17	7.66
Median	5.76	8.12
Standard Deviation	2.85	2.41
VC	46%	32%
Min	0.50	2.34
Max	12.32	13.42
N	27	28
CI	1.07	0.89
*p*-value	0.041*	

VC - variation coefficient; Min - minimum value; Max - maximum value; N - number of mean values (participants); CI - confidence interval

The results indicate that the groups are statistically different, G1 showed lower mandible shifting to the left when compared to G2.

Table 4 compares the groups as to mandible protrusion.

**Table 4:** Comparing mandible protrusion between GI and G2.

Protrusion	G1	G2
Mean	5.05	7,99
Median	5.03	8,53
Standard Deviation	2.50	2,09
VC	49%	26%
Min	0.32	3,43
Max	9.71	13,36
N	25	28
CI	0.98	0,77
*p-value*	<0.001*	

VC - variation coefficient; Min - minimum value; Max - maximum value; N - number of mean values (participants); CI - confidence interval

Results indicate that the groups are statistically different, and G1 presented less mandible protrusion than G2.

Because of the statistically significant difference between the groups in relation to left mandibular shifting, we carried out new analysis for G1 in order to check the influence of the paralyzed side in mandible shifting. To do that, we used the ANOVA tests and 0.05 of significance level.

As per shown on Table 5, there was no statistically significant difference concerning mandible lateralization, considering G1 subdivisions (facial paralysis on the right and left), in other words, the paralyzed side did not interfere on mandible movement.

**Table 5:** Comparison between the paralyzed hemifaces as to mandible lateralization.

Paralysis Side	Effect	SQ	DF	MQ	F	^*p*^
Right	Lateralization	5.87	1	5.870	0.459	0.504
	Error	332.34	26	12.782		
Left	Lateralization	4.61	1	4.612	0.653	0.427
	Error	169.60	24	7.067		

### DISCUSSION

Given the data we analyzed, we concluded that there is a significant mean difference between the groups concerning the maximum mouth opening results, left side lateralization and mandible protrusion. In these three variables we noticed that G1 always have results below those in G1. According to previous studies^19^, ^29^, ^30^, the normal values for mandible movements are: for maximum mouth opening (between 40mm and 60mm); mandibular lateralization for both sides (between 7mm and 11mm) and mandible protrusion value (between 7mm and 11mm), without distinction as far as gender and age are concerned. These values were confirmed for G2. Nonetheless, for G1, the lateralization and protrusion values are below normal values.

Mandible movements enable changes in the intraoral spaces, allowing for free movements of the tongue and of the soft tissue, determining many of the characteristics of mastication, swallowing and speech^21^. The proper TMJ functioning has a very positive effect on the stomatognathic functions and in that of the orofacial muscles in general. Notwithstanding, even with functional and/or structural changes, the orofacial functions are made feasible by means of adaptations, most of the times the person is not even aware of^30^. The evaluation of the mandibular range of motion is usually part of protocols which assess TMJ's integrity and functionality. Muscle and structural adaptations arising from numerous disorders of different etiologies may be responsible for the reduction in muscle elongation, which in its turn will impact mandible movement (opening, protrusion and lateralization)^19^, ^20^, ^21^, ^31^, ^32^, ^33^, ^34^.

Mandibular movement limitations may be caused by muscle disorders. When there is muscle pain, the patient tends to reduce activity in the muscles involved^19^. This reduction in muscle function may cause changes to them, such as atrophy because of lack of use, thus bringing about strength reduction, restriction in opening movements and shifts^21^.

More specifically, and according with the literature, the limitation in mandible opening may have many causing factors: muscle contraction for protection and spasm arising from the increase in metabolic degrading products caused by changed muscle activity, both situations aim at avoiding a feeling of discomfort (pain)^34^. Still, according to the literature, facial muscle pain is a condition which can be associated to vegetative changes such as absence or reduction in tearing, vascular changes, or changes to the co-contraction of adjacent muscles in the case of functional unbalances of the muscles, as it happens in Facial Paralysis^34^.

Although the results have pointed to significant differences between the groups only for left-side mandibular shifting, it is important to stress that for G1 the mean value of mandible shifting to the right is below the expected interval for normality, pointing to changes. Numerous studies point out that the chewing physiology involves mandible protrusion movements which enable food capture and movements with enable grinding and food pulverization^20^, ^30^. The reduction in mandibular range of motion in protrusion and lateralization, either because of muscle atrophy or secondary to pain, may cause changes or compensations in the execution of stomatognathic functions.

Even when the limitation in mandibular range of motion is muscular in origin, data from the present study suggests that the permanence of the functional unbalance may be a triggering and/or worsening factor concerning the structural changes in TMJ (e.g. joint disc shifting, joint pain)^24^,^26^,^34^.

Results indicate that the patients affected by Facial Paralysis have a significant reduction in mandibular range of motion when compared to the control group. Since mandibular range of motion is a TMD predictive factor, the results from the present study support the suggestion that we should add functional tests of the stomatognathic system, orofacial and TMJ function tests to the clinical assessment of facial paralysis.

### CONCLUSION

The present study enables us to educate those professionals involved in the diagnosis and treatment of Bell's Facial Paralysis that more attention should be given to orofacial muscle functional unbalances and their anatomical implications, as is the case of TMJ function.

A future study will be developed for the evaluation of the chewing muscles in individuals with idiopathic facial paralysis, which may help us better understand the functional implications of the TMJ biomechanical paralysis.

ATTACHMENT 1 Toledo, P. N. Efeito da terapia miofuncional em pacientes com paralisia facial de longa duração associada à aplicação de toxina botulínica. 2007. Thesis (PhD in Plastic Surgery) - Medical School of the University of São Paulo.

Date: ____/____/____ Examiner:_________________________

1 VOLUNTARY MOVEMENT RIGHT SIDE LEFT SIDE
FOREHEAD 0 1 2 0 1 2
EYELIDS 0 1 2 0 1 2
UPPER LIP ELEVATION 0 1 2 0 1 2
MOUTH OBLIQUE TRACTION 0 1 2 0 1 2
MOUTH HORIZONTAL TRACTION 0 1 2 0 1 2
LIP CLOSURE 0 1 2 0 1 2
LOWER LIP DEPRESSION 0 1 2 0 1 2
TOTAL __ __ __ __ __ __
2 INVOLUNTARY MOVEMENT RIGHT SIDE LEFT SIDE
BLINKING 0 1 2 0 1 2
SPEAKING 0 1 2 0 1 2
SMILE / LAUGH 0 1 2 0 1 2
TOTAL __ __ __ __ __ __
3 NEGATIVE FINDINGS RIGHT SIDE LEFT SIDE
DEFORMITY (REST) EYELIDS 0 -1 -2 0 -1 -2
DEFORMITY (REST) MOUTH 0 -1 -2 0 -1 -2
SYNKINESIA / HYPERTONIA 0 -1 -2 0 -1 -2
TOTAL __ __ __ __ __ __
TOTAL FINAL __ __ __ __ __ __
(0) ABSENT (1/-1) PARTIAL/MODERATE (2/-2) COMPLETE/MARKED
